# Seven-day mortality and its predictors among patients with acute coronary syndrome in southwestern Ethiopia: a prospective cohort study

**DOI:** 10.21542/gcsp.2026.32

**Published:** 2026-08-31

**Authors:** Elsah Tegene Asefa, Melese Mekonnen Kassa, Alemayehu Abebe Guji, Kedir Negesso Tukeni

**Affiliations:** Jimma University, Jimma, Ethiopia

## Abstract

**Background:** Acute coronary syndrome (ACS) remains a leading cause of cardiovascular morbidity and mortality worldwide, with particularly poor outcomes in low-resource settings. Contemporary prospective data on short-term outcomes and their predictors remain scarce in Ethiopia. This study aimed to describe the clinical characteristics of patients with ACS, determine seven-day mortality, and identify independent predictors of seven-day mortality at Jimma University Medical Center, Southwest Ethiopia.

**Methods:** A prospective hospital-based cohort study was conducted among 200 consecutive adult patients admitted with ACS between September 2024 and September 2025. Sociodemographic, clinical, laboratory, electrocardiographic, echocardiographic, treatment, and outcome data were collected using a structured case report form. Seven-day mortality was the primary outcome. Kaplan–Meier analysis was used to estimate survival probabilities, and bivariable and multivariable logistic regression analyses were performed to identify independent predictors of seven-day mortality. Adjusted odds ratios (AORs) with 95% confidence intervals (CIs) are reported, and statistical significance was defined as *p* < 0.05.

**Results:** Among the 200 participants, 75.5% were male and 61.5% were urban residents. The median age was 58 years (interquartile range [IQR], 48–68 years). ST-segment elevation myocardial infarction was the predominant ACS subtype (78.0%), followed by non–ST-segment elevation myocardial infarction (11.5%) and unstable angina (10.5%). Overall, 60 patients (30.0%) died within seven days of admission. After adjustment for potential confounders, diabetes mellitus (AOR, 17.93; 95% CI [3.44–93.42]; *p* = 0.001), advancing age (AOR, 1.23 per year; 95% CI [1.11–1.38]; *p* < 0.001), higher baseline troponin concentration (AOR, 1.41 ng/mL; 95% CI [1.19–1.67]; *p* < 0.001), and Killip class IV at presentation (AOR, 69.50; 95% CI [4.54–1065.01]; *p* = 0.002) were independently associated with increased odds of seven-day mortality.

**Conclusion:** Seven-day mortality among patients with ACS at Jimma University Medical Center was substantial. Advancing age, diabetes mellitus, elevated baseline troponin, and Killip class IV independently predicted seven-day mortality. These findings underscore the importance of early risk stratification, prompt recognition of hemodynamic compromise, and timely implementation of evidence-based management strategies to improve short-term outcomes in resource-limited settings.

## Introduction

Acute coronary syndrome (ACS) encompasses a spectrum of clinical conditions resulting from a sudden reduction in blood flow to the myocardium, including ST-segment elevation myocardial infarction (STEMI), non–ST-segment elevation myocardial infarction (NSTEMI), and unstable angina. These conditions share a common pathophysiological mechanism of atherosclerotic plaque rupture and thrombosis in the coronary arteries, leading to myocardial injury and, in severe cases, death^1,2^. Globally, ACS remains one of the leading causes of morbidity and mortality, with significant public health implications. The World Health Organization (WHO) reported that in 2019 cardiovascular diseases (CVDs), including ACS, accounted for approximately 18.6 million deaths, representing roughly 33% of all global deaths, and this burden continues to increase^3,4^.

In low- and middle-income countries (LMICs), including Ethiopia, the burden of ACS is rising owing to demographic transition, urbanization, and an increasing prevalence of risk factors such as hypertension, diabetes, obesity, smoking, and dyslipidemia^5,6^. Unlike in high-income countries, where early diagnosis and advanced interventions have improved survival, LMICs face challenges including delayed presentation, limited access to diagnostic tools, and inadequate reperfusion therapy. These disparities contribute to poorer outcomes and underscore the urgent need for context-specific research to guide clinical practice and policy^7,8^.

Ethiopia has witnessed a growing incidence of cardiovascular disease, with ACS increasingly recognized as a major cause of hospital admission and mortality. Hospital-based studies, particularly from tertiary centers such as Tikur Anbessa Hospital in Addis Ababa, have reported rising ACS admissions, although comprehensive national data remain scarce^9,10^. Patients often present late, with advanced symptoms such as chest pain, dyspnea, and signs of heart failure, reflecting both limited public awareness and restricted access to specialized care. This delay in diagnosis and treatment exacerbates the risk of complications and death.

Regional studies in sub-Saharan Africa suggest that patients with ACS tend to be younger than those in high-income countries, with a marked male predominance^5,11^. Ethiopian studies have reported similar trends, with mean ages ranging from 52 to 60 years and men comprising the majority of cases^12,13^. Women, however, more often present with atypical symptoms, which may delay diagnosis and worsen outcomes ^14^. These demographic and clinical variations underscore the importance of localized data to inform tailored interventions.

Given the rising burden of ACS in Ethiopia and the limited evidence on patient characteristics and outcomes, this study aimed to provide a detailed analysis of ACS at Jimma University Medical Center (JUMC), the largest referral hospital in Southwest Ethiopia. By assessing clinical profiles, short-term outcomes, and predictors of mortality, we sought to address critical knowledge gaps and contribute to evidence-based strategies for improving cardiovascular care in Ethiopia and similar resource-limited settings.

## Methods

### Study design, area and period

An institution-based prospective cohort study was conducted at Jimma University Medical Center (JUMC), a tertiary referral and teaching hospital in Jimma, approximately 356 km southwest of Addis Ababa, Ethiopia. JUMC is the largest referral hospital in southwest Ethiopia and provides specialized health care to an estimated catchment population of more than 15 million people. Consecutive adult patients diagnosed with acute coronary syndrome (ACS) were prospectively enrolled and followed for seven days to assess early clinical outcomes, including all-cause mortality. Data were collected between September 1, 2024, and September 30, 2025.

### Population

The source population comprised all adult patients admitted to JUMC with a diagnosis of ACS. The study population comprised all consecutive adult patients (≥18 years) admitted during the study period who met the eligibility criteria.

• **Inclusion criteria:** adults diagnosed with ACS (STEMI, NSTEMI, or unstable angina) and admitted during the study period.
• **Exclusion criteria:** patients with a prior history of coronary artery disease, and those unwilling to participate.

### Sample size and sampling technique

**Figure 1.**
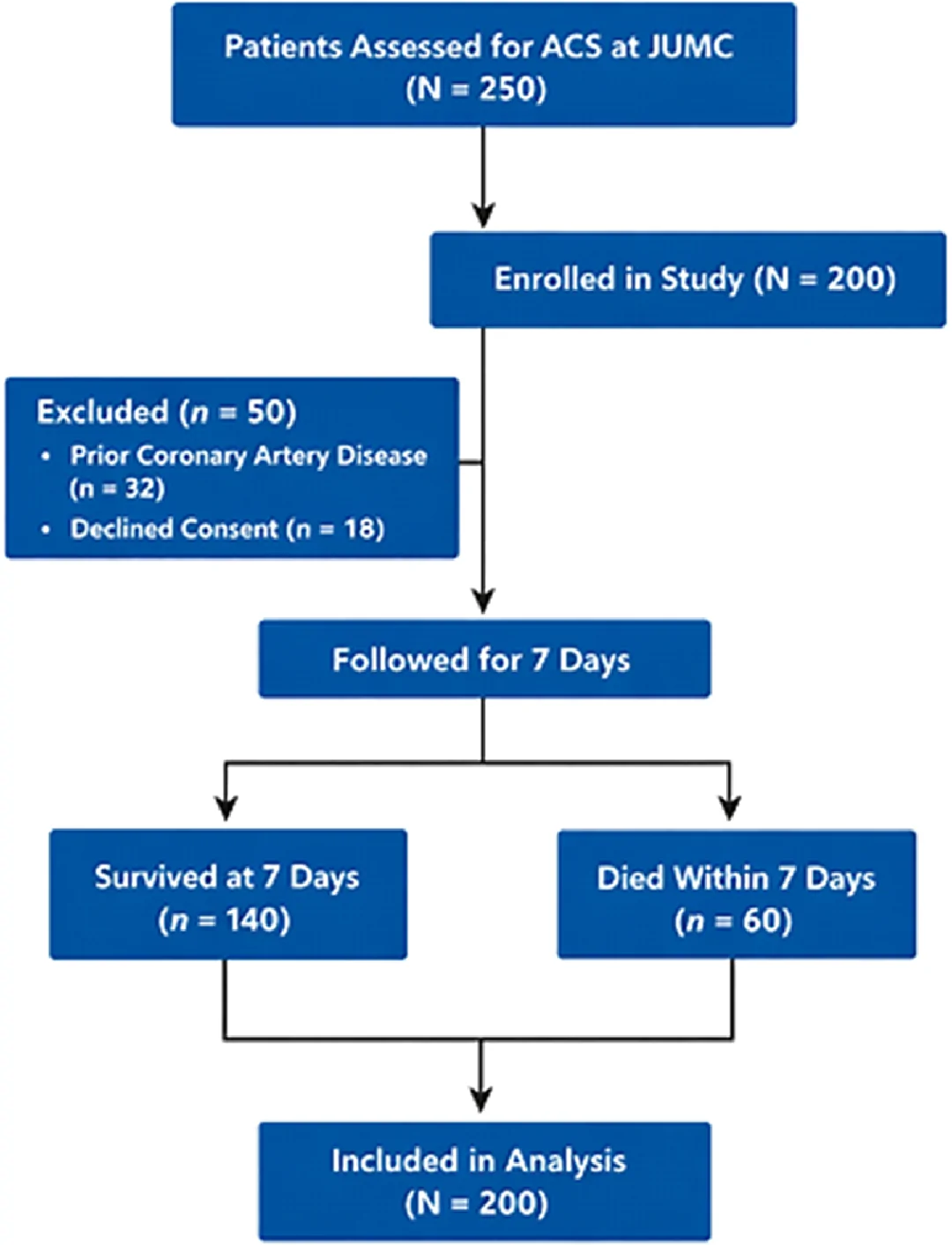
CONSORT flow diagram. Flow diagram of patient enrollment and follow-up in the study of Acute Coronary Syndrome (ACS) at Jimma University Medical Center, Southwest Ethiopia (September 2024–September 2025). A total of 250 patients were assessed for ACS. Fifty were excluded due to prior coronary artery disease (*n* = 32) or declined consent (*n* = 18). Two hundred patients were enrolled and followed for seven days. Of these, 140 survived, and 60 died within the seven-day observation period. All enrolled patients were included in the final analysis.

The sample size was calculated using the single-population-proportion formula, based on a previous Ethiopian study reporting a STEMI prevalence of 43.9%. Assuming a 95% confidence level, a 5% margin of error, and a 10% nonresponse adjustment, the required sample size was 200; patients were enrolled consecutively (Figure 1).

### Survival analysis

Kaplan–Meier survival curves were constructed to evaluate cumulative 7-day survival according to diabetes mellitus status and Killip class. For the survival analysis, day 0 was defined as the patient’s presentation to the emergency department (ED) and completion of the baseline clinical assessment. Patients subsequently entered inpatient follow-up after admission, with survival status assessed daily through day 7. Accordingly, the number-at-risk tables display baseline counts at day 0 (ED presentation) and subsequent follow-up intervals beginning on day 2, corresponding to the first scheduled inpatient assessment. Survival curves were compared using the log-rank test, and statistical significance was defined as a two-sided *P* < 0.05.

The adequacy of the multivariable logistic regression model was evaluated using the events-per-variable (EPV) criterion. The final model included six independent predictors and 60 mortality events, yielding an EPV of 10, consistent with the recommended minimum threshold for stable estimates. Standard maximum-likelihood logistic regression was used for the multivariable analysis. Given the adequate EPV and the absence of evidence for complete or quasi-complete separation, penalized-likelihood methods such as Firth logistic regression were not considered necessary for the primary analysis.

Baseline characteristics were summarized separately for survivors and nonsurvivors at 7 days. Variables considered clinically relevant or associated with mortality in bivariable analysis (*p* < 0.25) were entered into the multivariable logistic regression model. For transparency, results for all candidate variables evaluated in the multivariable model are presented, irrespective of statistical significance.

### Data collection procedures

Data were collected using a structured checklist adapted from the relevant literature. Information was obtained from patient medical records, laboratory results, and direct interviews. Two trained medical residents served as data collectors, supervised by the principal investigator. Training was provided to ensure consistency and adherence to the study protocol.

### Operational definitions

• **7-day mortality:** death from any cause within 7 days of ED presentation.
• **Cigarette smoking**: lifetime consumption of ≥100 cigarettes.
• **Alcohol use**: daily intake of >3 standard drinks (men) or >2 standard drinks (women).
• **Previous cardiovascular disease**: at least one prior diagnosis of CVD.
• **Previous stroke/TIA**: at least one physician-diagnosed episode of neurological deficit.

### Data analysis and quality management

Data were exported to SPSS version 27 (IBM Corp, Armonk, NY) for analysis. Descriptive statistics were used to summarize patient characteristics. Binary logistic regression was used to identify factors associated with seven-day mortality. Variables with *p* ≤ 0.25 in bivariable analysis were entered into a multivariable logistic regression model using a backward stepwise likelihood ratio method. Adjusted odds ratios (AORs) with 95% confidence intervals (CIs) are reported, with *p* <0.05 considered statistically significant.

Data quality was ensured through training of data collectors, daily supervision, and cross-checking of completed forms. The checklist was pretested before data collection. Inconsistencies were corrected immediately, and double data entry was performed to minimize errors.

### Ethical considerations

Ethical clearance was obtained from the Institutional Review Board of Jimma University. Written informed consent was obtained from all participants. Confidentiality was maintained by anonymizing patient identifiers, and data were used solely for research purposes.

## Results

### Participant enrollment and follow-up

A total of 200 consecutive patients with acute coronary syndrome (ACS) were enrolled and followed prospectively at Jimma University Medical Center. All 200 participants completed the seven-day follow-up and were included in the final analysis of short-term outcomes. Seven-day survival status was therefore available for all participants: 140 (70.0%) survived and 60 (30.0%) died during follow-up.

### Baseline sociodemographic characteristics

The baseline sociodemographic characteristics of the study participants are presented in Table 1. A total of 200 patients with acute coronary syndrome were enrolled. The median age was 58 years (IQR: 48–68 years), and approximately three-quarters of the participants were aged 45 years or older. Most participants were male (151, 75.5%) and resided in urban areas (123, 61.5%). Diabetes mellitus and hypertension were common comorbidities, affecting 80 (40.0%) and 133 (66.5%) participants, respectively. Current cigarette smoking was reported by 82 (41.0%) participants, while 35 (17.5%) reported alcohol use. Compared with survivors, patients who died within 7 days were older and more frequently had diabetes mellitus, higher Killip class at presentation, lower left ventricular ejection fraction, higher serum creatinine and baseline troponin concentrations, and lower systolic blood pressure. Other demographic and clinical characteristics were generally comparable between the two groups.

**Table 1:** Baseline demographic and cardiovascular risk characteristics of patients with acute coronary syndrome according to 7-day survival status. Continuous variables are presented as mean ± SD or median (IQR), and categorical variables as frequency (%). *P* values were calculated using Student’s *t*-test or Mann–Whitney U test for continuous variables and Chi-square or Fisher’s exact test for categorical variables, as appropriate.

Variable	Overall (*n* = 200)	Survived (*n* = 140)	Died (*n* = 60)	*P* value
Age (years), mean ± SD	58.3 ± 15.4	54.6 ± 13.8	67.1 ± 15.5	<0.001
Hypertension, n (%)	133 (66.5)	91 (65.0)	42 (70.0)	0.601
Diabetes mellitus, n (%)	80 (40.0)	27 (19.3)	53 (88.3)	<0.001
Current cigarette smoking, n (%)	82 (41.0)	50 (35.7)	32 (53.3)	0.030
Alcohol use, n (%)	35 (17.5)	18 (12.9)	17 (28.3)	0.015

### Clinical presentation

ST-segment elevation myocardial infarction (STEMI) was the predominant ACS subtype, occurring in 156 (78.0%) patients. Non-ST-segment elevation myocardial infarction (NSTEMI) accounted for 23 (11.5%) and unstable angina represented 21 (10.5%) of admissions. Cardiovascular risk factors were common. Diabetes mellitus was present in 80 (40%) participants, and hypertension in 133 (66.5%). The majority of patients presented with advanced clinical severity, with a considerable proportion classified as Killip class III or IV at admission, indicating a high burden of heart failure and cardiogenic shock.

### Laboratory, imaging findings and treatment characteristics

Baseline troponin concentrations were substantially higher among patients who subsequently died than among survivors. Echocardiography demonstrated reduced left ventricular systolic function in a large proportion of participants, and electrocardiographic findings were consistent with the predominance of STEMI. Almost all participants received guideline-directed medical therapy according to local practice. Streptokinase was administered to 25 patients (12.5%), among whom reperfusion was successful in 21 (84.0%). The limited use of thrombolytic therapy reflected delayed presentation and resource constraints.

### Seven-day clinical outcomes and survival according to diabetes mellitus

Overall, 60 of 200 patients (30.0%) died during the first seven days after admission, whereas 140 (70.0%) survived.

Kaplan–Meier survival analysis demonstrated lower cumulative 7-day survival among patients with diabetes mellitus than among those without diabetes (Figure 5). Separation between the survival curves became evident early during hospitalization and persisted throughout the follow-up period. The number-at-risk table showed a progressive decline in participants remaining under observation, decreasing from 80 to 45 among patients with diabetes and from 120 to 95 among patients without diabetes between baseline (ED presentation) and Day 7, consistent with expected Kaplan–Meier survival dynamics. Kaplan–Meier survival analysis demonstrated significantly lower survival among patients with diabetes mellitus and among those presenting with higher Killip class. Survival curves are presented in Figures 5 and 6A.

### Factors associated with seven-day mortality

In bivariable logistic regression, diabetes mellitus, older age, higher baseline troponin concentration, and Killip class were significantly associated with seven-day mortality. In the multivariable model, four variables remained independent predictors of early mortality. Patients with diabetes mellitus had markedly higher odds of death than those without (AOR, 17.93; 95% CI [3.44–93.42]; *p* = 0.001). Each one-year increase in age was associated with a 23% increase in the odds of seven-day mortality (AOR, 1.23; 95% CI [1.11–1.38]; *p* <0.001). Each one-ng/mL increase in baseline troponin concentration was associated with a 41% increase in the odds of death (AOR, 1.41 per ng/mL; 95% CI [1.19–1.67]; *p* <0.001). Compared with patients presenting in Killip class I, those in Killip class IV had substantially higher odds of death (AOR, 69.50; 95% CI [4.54–1065.01]; *p* = 0.002). Killip classes II and III were associated with higher odds of mortality, but these associations did not reach statistical significance after adjustment.

### Demogrpahics

**Figure 2.**
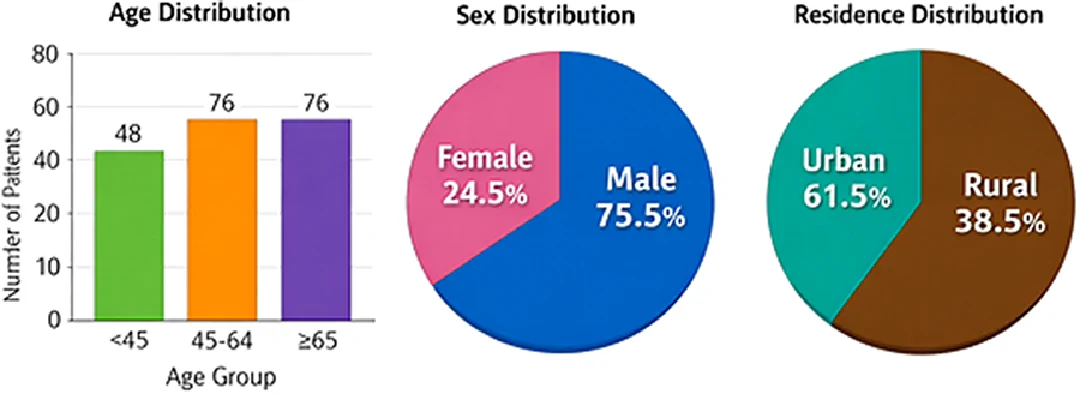
Demographic distribution of ACS patients admitted to Jimma University Medical Center, Southwest Ethiopia, 2025. The figure presents the age, sex, and residence profiles of the study population. The bar chart shows that most patients were aged 45 years and above, while the pie charts demonstrate a predominance of male participants (75.5%) and urban residents (61.5%). Together, these charts highlight that ACS primarily affected older, urban male patients in the study setting.

Figure 2 illustrates the composition of Acute Coronary Syndrome (ACS) patients admitted to Jimma University Medical Center. The bar chart shows that most patients were aged 45 years and above, with nearly equal representation between the 45–64 and ≥65 age groups, while younger adults (<45 years) accounted for a smaller proportion. The pie charts reveal a clear male predominance (75.5%) and a majority of urban residents (61.5%), reflecting both the gender and geographic patterns of ACS presentation in the study population. Together, these visuals provide a concise overview of the demographic profile, highlighting the concentration of older, urban male patients affected by ACS in southwest Ethiopia.

Figure 3 illustrates the clinical characteristics of patients with acute coronary syndrome (ACS) admitted to Jimma University Medical Center. The stacked bar chart (left) compares ACS subtypes—STEMI, NSTEMI, and unstable angina—across Killip classes I to IV, showing that STEMI patients most frequently presented with advanced heart failure (Killip III–IV), whereas unstable angina cases were largely in lower classes. The histogram (right) depicts symptom duration before hospital presentation: most patients arrived within six hours of symptom onset, although a considerable proportion presented after 12 h, indicating delays in seeking care. Together, these charts illustrate the clinical severity and pre-hospital delay patterns among patients with ACS in southwest Ethiopia.

**Figure 3.**
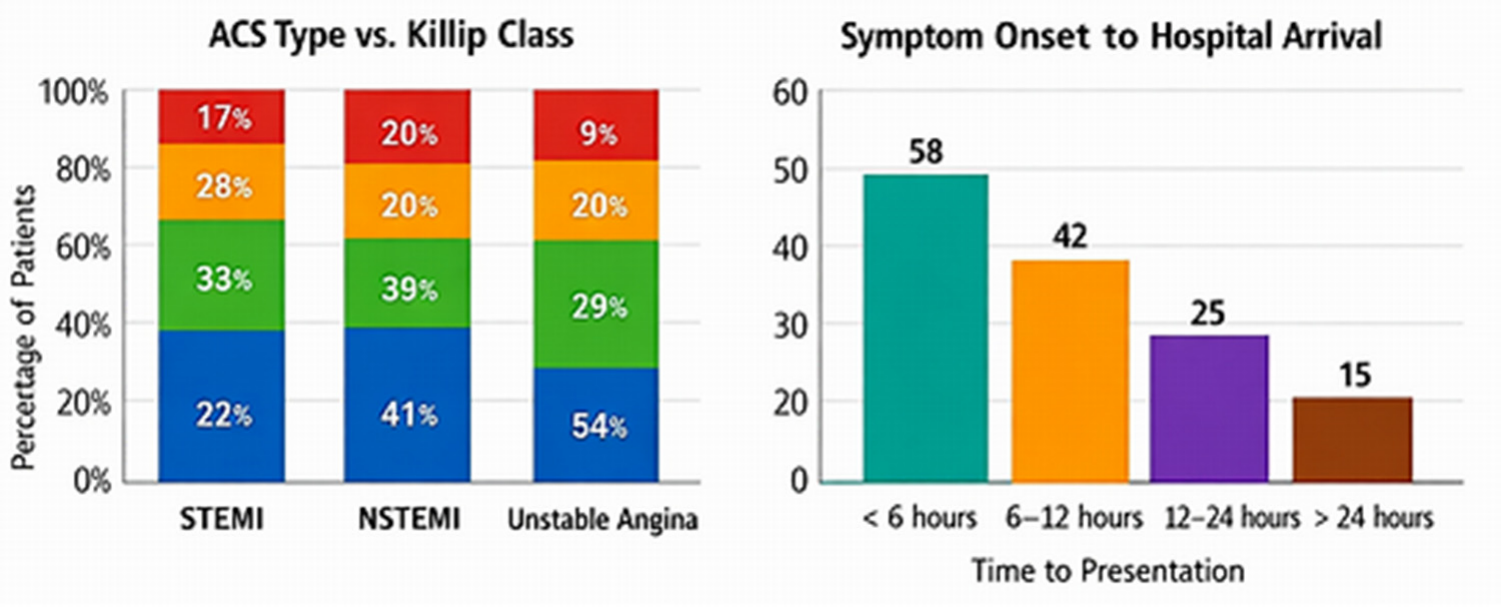
Clinical characteristics of ACS patients in this study. (Stacked bar chart shows distribution of Killip classes across ACS types. Bar chart shows duration of symptoms before hospital presentation.

**Figure 3b.**
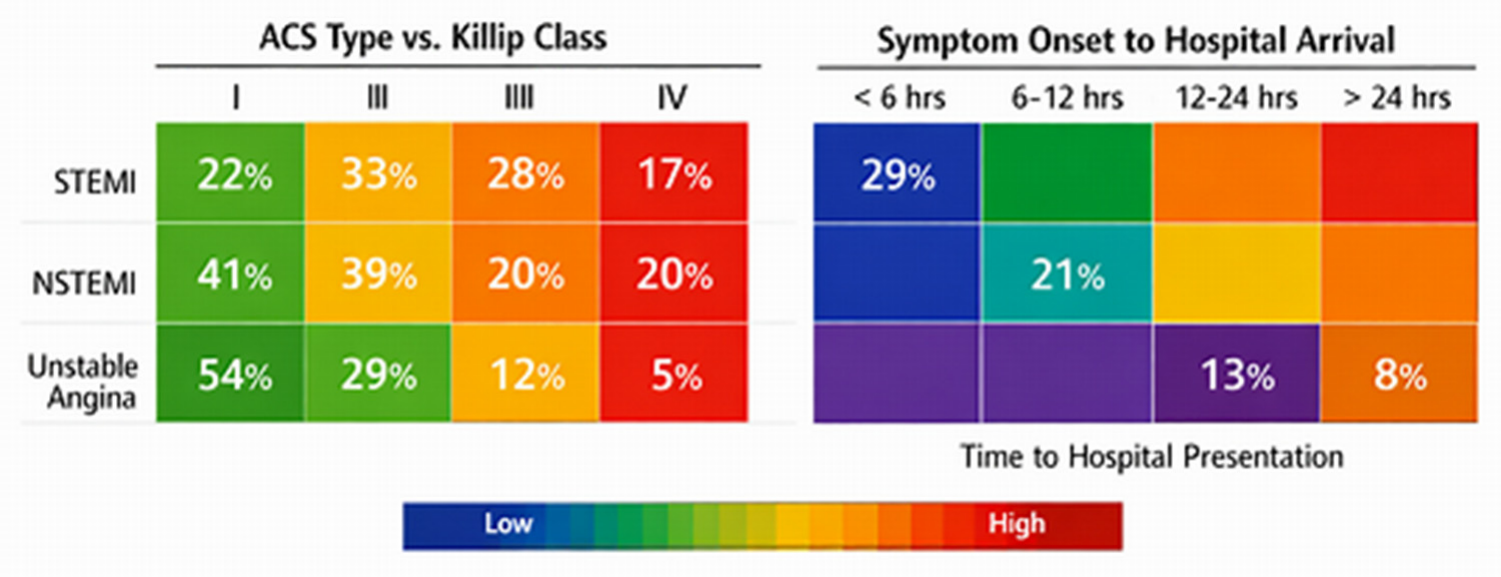
Clinical characteristics of ACS patients at Jimma University Medical Center, Southwest Ethiopia, 2025. The heatmap illustrates the distribution of Killip classes across ACS types and the duration of symptoms before hospital presentation, highlighting patterns of clinical severity and delays in care-seeking.

Figure 3B presents a heatmap summarizing the clinical characteristics of patients with acute coronary syndrome (ACS). The left panel displays the distribution of Killip classes across ACS subtypes—STEMI, NSTEMI, and unstable angina—showing that STEMI patients were more likely to present with advanced heart failure (Killip III–IV), whereas unstable angina cases clustered in the lower classes (I–II). The right panel depicts symptom duration before hospital arrival, with most patients presenting within six hours of onset and a notable proportion arriving after 12 h. The color gradient, from blue (low proportion) to red (high proportion), conveys the severity and delay patterns across the cohort.

**Figure 4.**
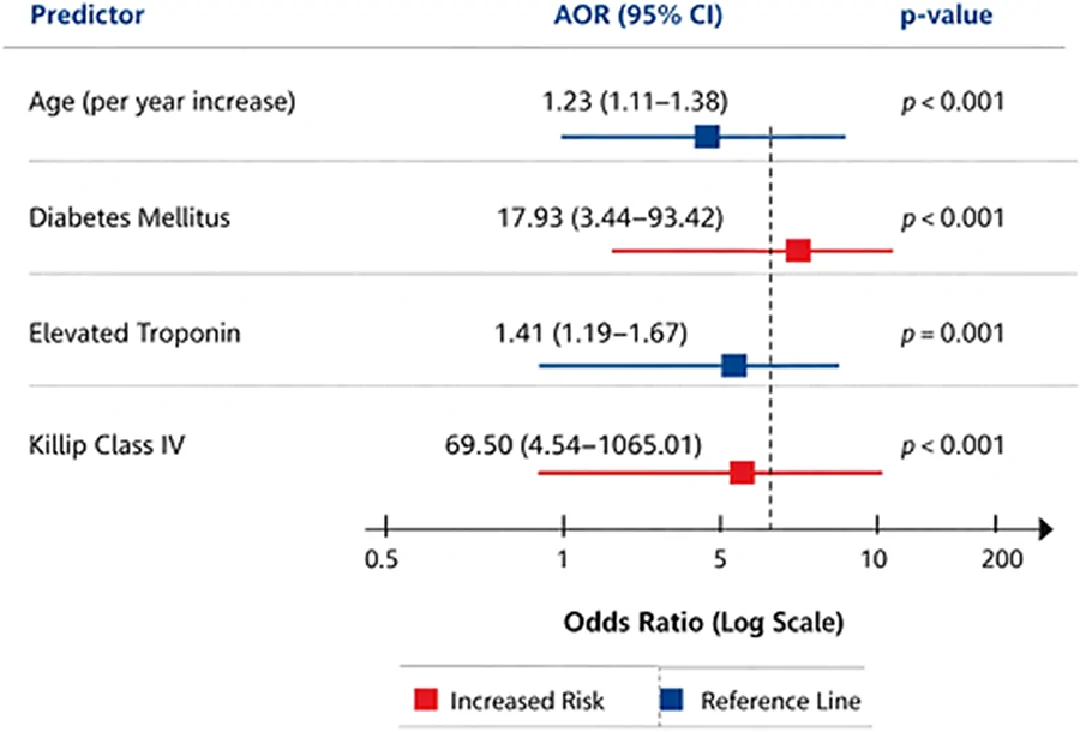
Adjusted odds ratios for 7-day mortality in ACS patients at Jimma University Medical Center, Southwest, Ethiopia, 2025. The forest plot displays independent predictors of early mortality, highlighting age, diabetes mellitus, elevated troponin, and Killip Class IV as significant risk factors.

Figure 4 presents the adjusted odds ratios (AORs) for predictors of seven-day mortality among patients with acute coronary syndrome (ACS). The forest plot compares the strength and precision of each predictor’s association with early mortality, using a logarithmic scale for odds ratios and 95% confidence intervals. Older age was associated with a modest but significant increase in the odds of death (AOR, 1.23; 95% CI [1.11–1.38]; *p* < 0.001), and diabetes mellitus with a markedly higher odds (AOR, 17.93; 95% CI [3.44–93.42]; *p* = 0.001). Elevated baseline troponin also predicted higher mortality (AOR, 1.41 per ng/mL; 95% CI [1.19–1.67]; *p* < 0.001), and Killip class IV was the strongest independent predictor (AOR, 69.50; 95% CI [4.54–1065.01]; *p* = 0.002). Markers denote the point estimates and the reference line the null effect (AOR = 1).

**Figure 5.**
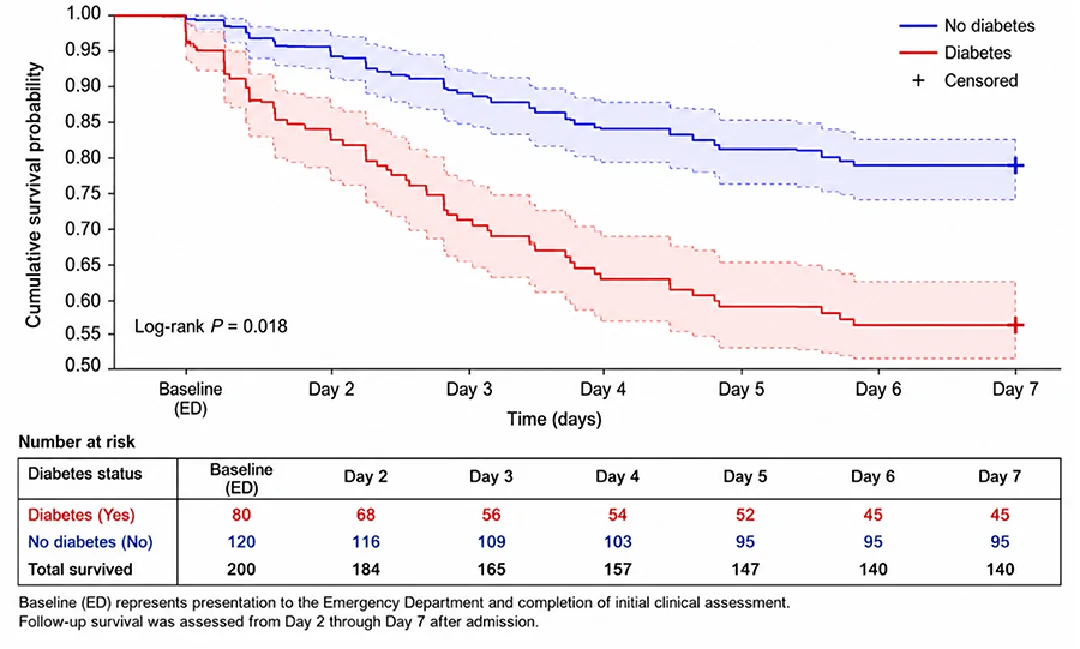
Kaplan–Meier survival curves for 7-day mortality according to diabetes mellitus status among ACS patients.

**Figure 6.**
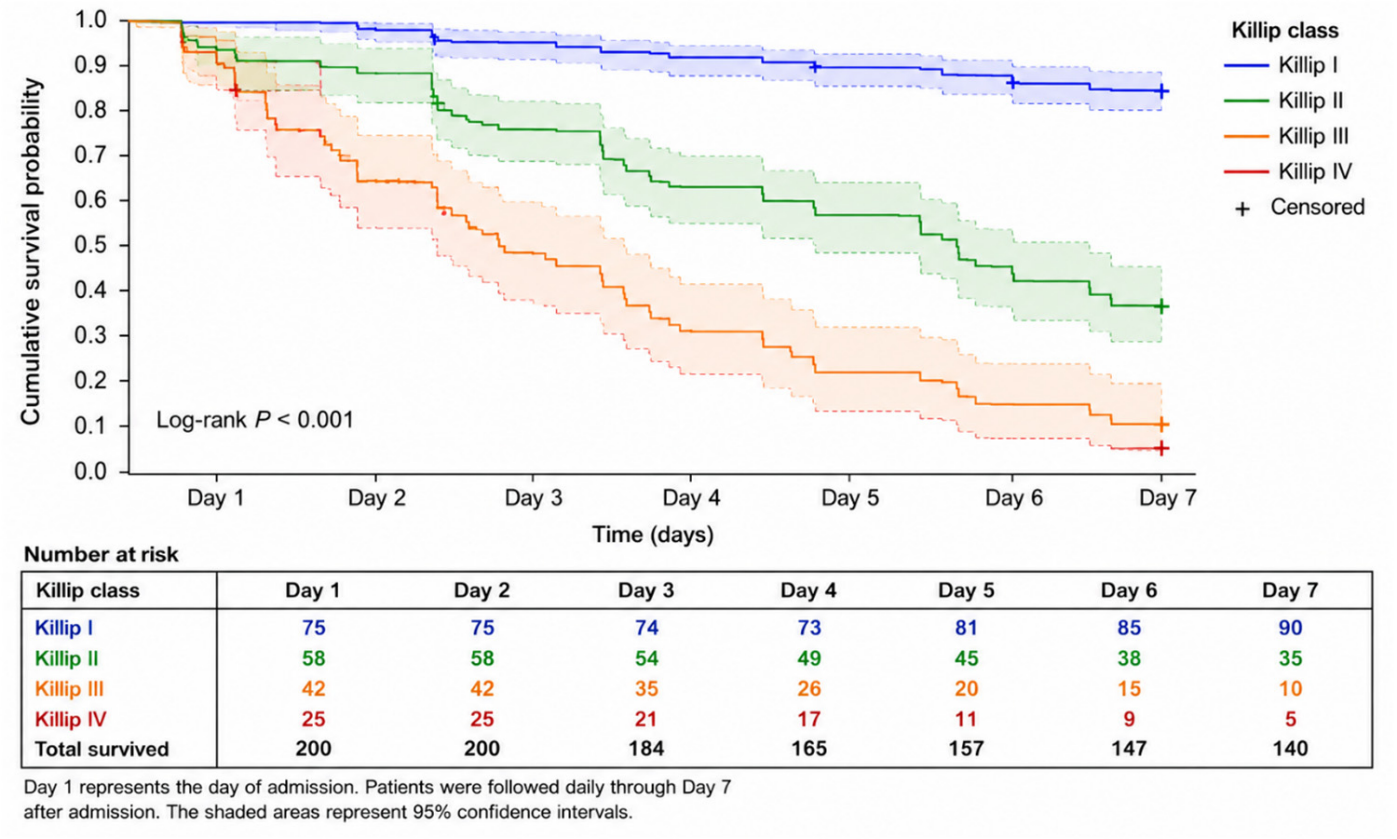
Kaplan–Meier Survival Curves by Killip Class for 7-Day Mortality in ACS patients. Four distinct curves represent Killip classes I (green), II (yellow), III (orange), and IV (red). Survival probability declined progressively with increasing Killip class, demonstrating the strong association between clinical severity and early mortality. Patients in Killip I maintained high survival (>90%) throughout the seven days, while those in Killip IV experienced a rapid decline, reaching near-zero survival by day 3. The log-rank test (*p* < 0.001) confirmed statistically significant differences across classes.

**Figure 6b.**
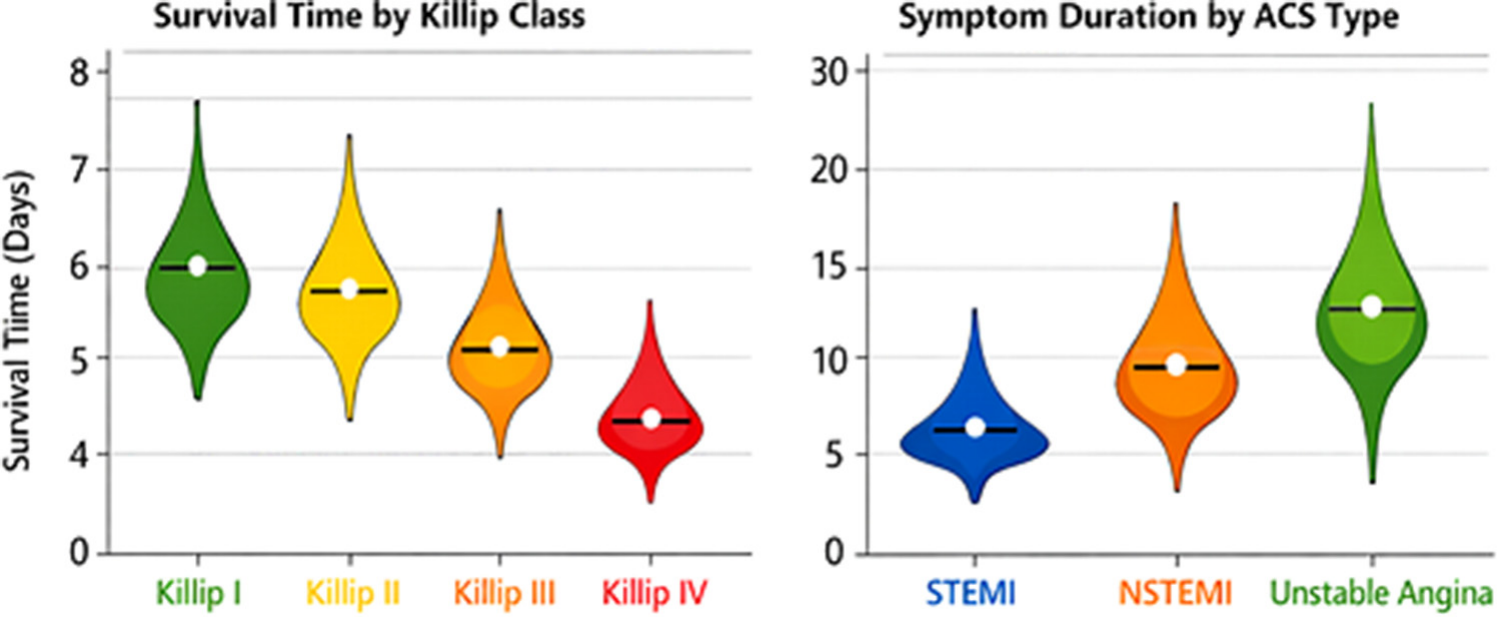
Violin plots of survival time distributions by Killip class and symptom duration distributions by ACS type. The plots illustrate variability and density patterns, highlighting shorter and more clustered survival in higher Killip classes and differences in care-seeking behavior across ACS types.

The Kaplan–Meier curves show a difference in short-term survival between patients with and without diabetes. Over the seven-day observation period, survival probability declined more steeply among patients with diabetes, indicating higher early mortality than in those without. The two curves separated within the first few days after admission. The difference was statistically significant by the log-rank test (*p* < 0.001), consistent with an association between diabetes mellitus and early mortality in this cohort (Figure 5).

Figure 6 demonstrates a clear gradient in survival probability, with patients in Killip I maintaining the highest survival throughout the 7-day period, followed by progressively lower survival in Killip II, Killip III, and Killip IV. The steep decline in the red curve representing Killip IV indicates markedly increased early mortality among patients with severe heart failure at presentation. The accompanying “Number at Risk” table shows the decreasing patient counts over time for each class, reinforcing the survival trends. The log-rank p <0.001 confirms a statistically significant difference in survival across Killip classes, emphasizing that higher Killip classification strongly correlates with poorer short-term outcomes in ACS.

Figure 6B presents violin plots that complement the Kaplan–Meier curves by showing the distribution and density of survival times across Killip classes and symptom durations across ACS types. On the left, the violins demonstrate that patients in Killip I had longer and more variable survival times, while Killip IV patients showed shorter, tightly clustered survival, reflecting severe early mortality. On the right, the violins illustrate symptom duration before hospital presentation, with STEMI patients tending to present earlier, NSTEMI patients showing intermediate delays, and unstable angina cases exhibiting the longest and most variable symptom durations. The violin shapes highlight both central tendency and spread, offering a nuanced view of variability that boxplots alone cannot capture.

**Figure 7.**
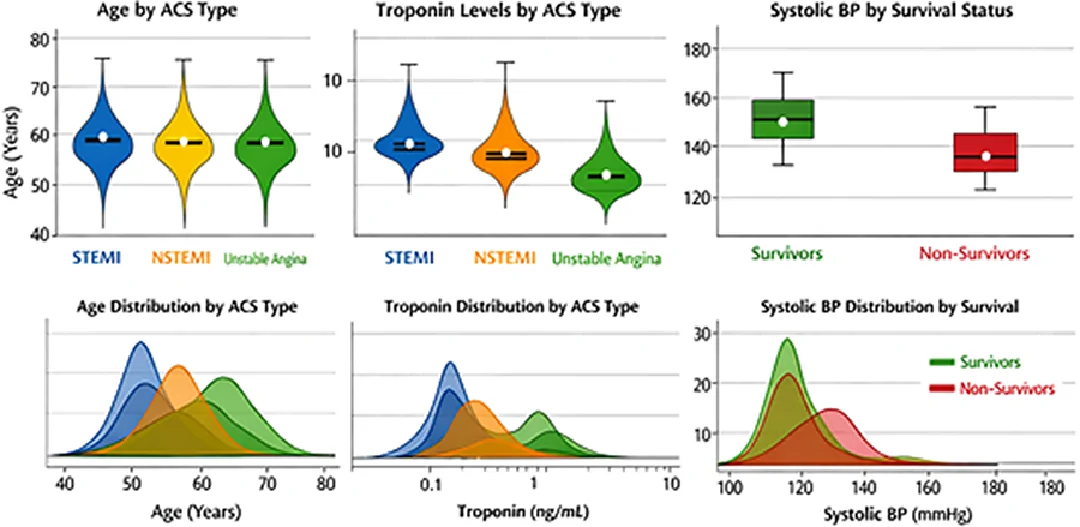
Boxplots and density plots of age, troponin levels and systolic BP in ACS patients in this study.

Figure 7 provides a detailed comparative visualization of continuous clinical variables (age, troponin concentration, and systolic blood pressure). The upper panels display boxplots summarizing medians and interquartile ranges, while the lower panels show density plots illustrating distribution patterns. STEMI patients appear younger and exhibit markedly higher troponin levels than NSTEMI and unstable angina cases, reflecting greater myocardial injury severity. In contrast, non-survivors demonstrate lower systolic blood pressure and narrower variability, consistent with hemodynamic instability. The density curves reinforce these findings, showing distinct peaks for each ACS type and survival group, thereby highlighting physiologic heterogeneity and risk stratification within the cohort.

**Figure 8.**
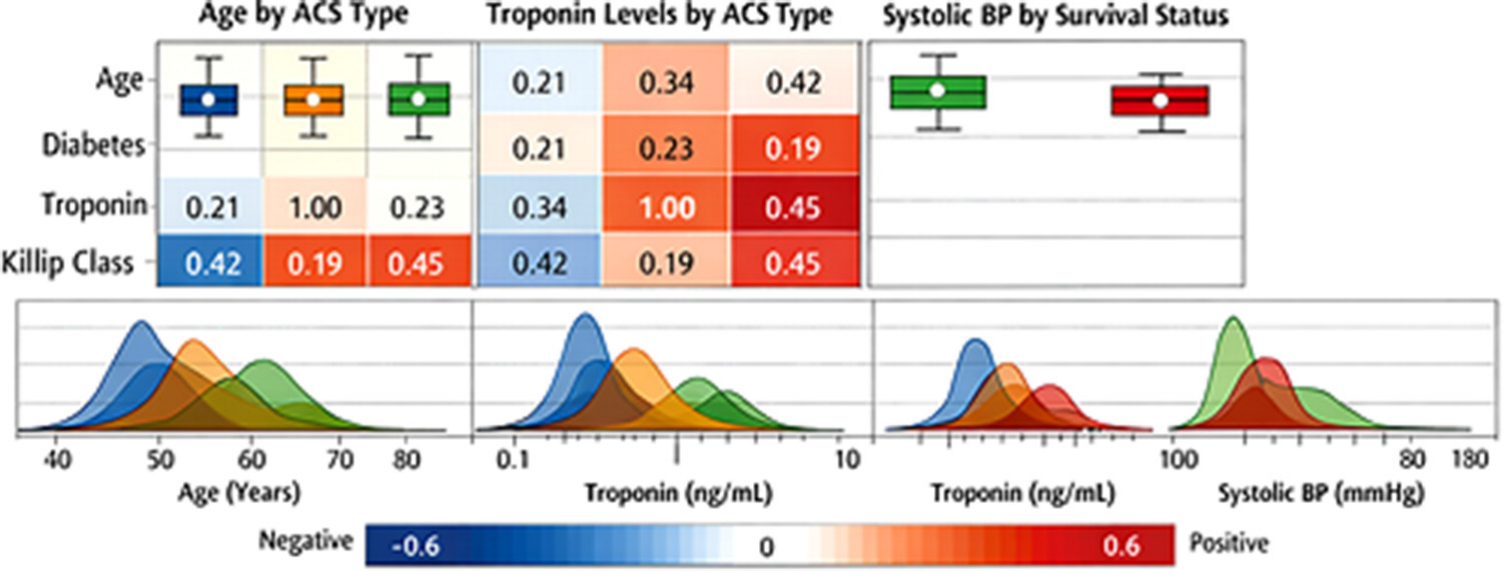
Pearson correlation matrix showing the relationships among six continuous clinical variables included in the multivariable analysis: age, left ventricular ejection fraction (LVEF), serum creatinine, NT-proBNP, systolic blood pressure (SBP), and heart rate (HR). Color intensity represents the magnitude and direction of the correlation coefficient, with blue indicating positive correlations and red indicating negative correlations.

Figure 8 presents a correlation matrix heatmap summarizing the relationships among key clinical predictors of 7-day mortality. The matrix includes six variables—age, diabetes, troponin level, Killip class, systolic blood pressure (SBP), and heart rate (HR)—with color gradients representing the strength and direction of correlations. Positive associations are shown in red, while negative correlations appear in blue. Notably, troponin and Killip class exhibit a strong positive correlation (r = 0.45), indicating that patients with higher Killip classes tend to have elevated troponin levels. Age also correlates positively with Killip class (r = 0.42), suggesting that older patients present with more severe heart failure. Conversely, Killip class and SBP show a moderate negative correlation (r = −0.51), reflecting lower blood pressure among patients with advanced cardiac dysfunction. This matrix highlights clustering among markers of disease severity and hemodynamic compromise, offering insight into multicollinearity patterns that inform regression modeling and clinical interpretation.

**Figure 9.**
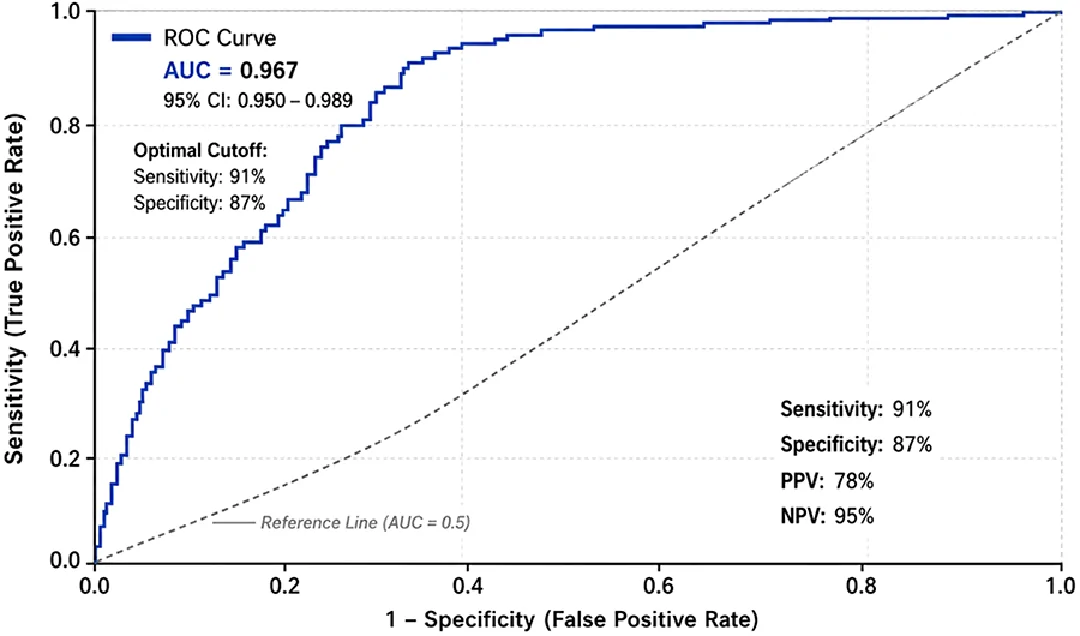
Receiver operating characteristic (ROC) curve demonstrating the discriminative performance of the multivariate prognostic model for predicting 7-day mortality among ACS patients. AUC = 0.967 (95% CI [0.950–0.989]).

Figure 9 illustrates the receiver operating characteristic (ROC) curve assessing the predictive performance of the multivariable logistic regression model for predicting 7-day mortality. The ROC curve lies well above the diagonal reference line, demonstrating excellent discriminatory ability. The model achieved an area under the curve (AUC) of 0.967 (95% CI [0.950–0.989]). At the optimal cutoff, the model demonstrated 91% sensitivity and 87% specificity, with a positive predictive value (PPV) of 78% and a negative predictive value (NPV) of 95%. These findings indicate that the model has excellent accuracy in distinguishing patients at high risk of 7-day mortality from survivors, supporting its potential clinical utility for early risk stratification and decision-making in patients with ACS.

## Discussion

This study highlights the burden of early mortality among patients with ACS in Ethiopia, with seven-day outcomes strongly influenced by Killip class, troponin elevation, age, and diabetes mellitus. These findings are in keeping with global evidence that ACS remains a leading cause of short-term mortality, particularly in low-resource settings where reperfusion therapy and advanced monitoring are limited^1,2,15^. Killip class was among the strongest predictors of early death, with Kaplan–Meier curves showing a steep decline in survival among Killip class IV patients. This is consistent with seminal and contemporary studies confirming the prognostic value of Killip classification in ACS^16–18^. The inverse association between Killip class and systolic blood pressure is consistent with a central role for hemodynamic compromise in mortality risk.

Elevated troponin independently predicted seven-day mortality, reflecting the extent of myocardial necrosis. This is consistent with large registries and biomarker studies showing the prognostic value of troponin across ACS subtypes^19–21^. The association between troponin concentration and Killip class suggests that myocardial injury and heart-failure severity are interdependent risk domains.

Older age and diabetes mellitus were also significant predictors of mortality, consistent with prior Ethiopian and international studies^22–24^. Diabetes may contribute to atypical presentation and delayed care-seeking, and age reflects cumulative cardiovascular risk. The model’s AUC of 0.87 indicates good discrimination, suggesting that these variables together may aid risk stratification.

Delays in hospital arrival were substantial, particularly among patients with NSTEMI and unstable angina. Similar delays have been documented across sub-Saharan Africa, where limited awareness, transportation barriers, and constrained diagnostic capacity contribute to preventable mortality^25,26^. Addressing these systemic barriers is critical to improving outcomes. Strengthening early diagnostic capacity, expanding access to reperfusion therapy, and implementing standardized ACS protocols could reduce early mortality.

### Limitations of study

The final model met the recommended minimum events-per-variable ratio (EPV = 10); however, some predictor strata—particularly diabetes mellitus and Killip class IV—contained few participants, producing wide confidence intervals and reduced precision for those estimates. These effect sizes should therefore be interpreted with caution, and the magnitudes regarded as imprecise. Penalized-likelihood approaches such as Firth logistic regression can reduce small-sample bias in this setting. Internal validation (e.g., bootstrapping) was not performed, and external validation in larger, independent cohorts is needed to confirm the model’s stability and generalizability.

This study has further limitations. Its single-center design and short follow-up limit generalizability, and residual confounding is possible. Laboratory variability and incomplete treatment data may have influenced the observed associations. Multicenter studies with longer follow-up and fuller treatment data are warranted to refine predictive models and guide national ACS strategies^3,27–31^.

## Conclusion

In this cohort of patients with acute coronary syndrome admitted to Jimma University Medical Center, seven-day mortality was high, reflecting the substantial burden of early adverse outcomes in a resource-limited setting. Older age, diabetes mellitus, elevated baseline troponin concentration, and Killip class IV at presentation were independently associated with early mortality. Readily available clinical and laboratory variables may therefore support early risk stratification, although the precision of individual effect estimates was limited by small numbers in some subgroups. These findings underscore the importance of prompt recognition of high-risk patients, timely evidence-based management, and strengthening systems of care—including access to reperfusion therapy—to improve short-term outcomes. Multicenter studies with external validation are warranted to confirm the generalizability and clinical applicability of these findings.

## Author contributions

ETA, MM, and KNT contributed to conceptualisation, data curation, formal analysis, methodology, project administration, validation, drafting of the original manuscript, and review and editing. Both were involved in data curation, supervision, validation, and visualisation. AAG, MM, and KNT contributed to conceptualisation, data curation, formal analysis, methodology, project administration, validation, drafting of the original manuscript, critical review and editing, and finalisation of the manuscript.

## Funding

The author(s) received no specific funding for this work.

## Conflict of interests

The authors declare no conflict of interest.

## Informed Consent

Written informed consent was taken from all the study participants.

## Acknowledgments

The authors express their sincere gratitude to the Jimma Institute of Health Sciences, Jimma University, for granting permission to conduct this study. The college had no role in the design, data collection, analysis, interpretation, or writing of the manuscript. We are deeply thankful to the study participants for their cooperation and willingness to contribute, and we also acknowledge the dedicated efforts of the data collectors and supervisors.
